# Supplementary material for: From yield stress to elastic instabilities: Tuning the extensional behavior of elastoviscoplastic fluids
Source: PNAS Nexus. 2024 Jun 5;3(6):pgae227. doi: 10.1093/pnasnexus/pgae227 (PMC11192060; doi:10.1093/pnasnexus/pgae227)
Supplement: pgae227_Supplementary_Data [file pgae227_supplementary_data.zip › PNASNEXUS-PNASNEXUS-2024-00345R-s01.pdf]

PNAS nexus

Supporting Information for

From yield stress to elastic instabilities: tuning the extensional behavior of elastoviscoplastic fluids

Mohamed S. Abdelgawad, Simon J. Haward, Amy Q. Shen and Marco E. Rosti

Marco E. Rosti
E-mail: marco.rosti@oist.jp

This PDF file includes:

Supporting text
Figs. S1 to S11
Table S1
Legend for Movie S1
SI References

Other supporting materials for this manuscript include the following:

Movie S1

Supporting Information Text

Large-amplitude oscillatory shear (LAOS) measurements

Fourier Transform (FT) rheology allows for the quantification of nonlinearities in LAOS by converting the time-domain stress response into a frequency spectrum, identifying intensities and phases of higher harmonics. In particular, given a sinusoidal strain input, $\gamma(t) = \gamma_0 \sin(\omega t)$, the stress response is expressed as

$$\tau(t;\omega,\gamma_0) = \gamma_0 \sum_{n:odd} \left\{ G'_n(\omega,\gamma_0)\sin(n\omega t) + G''_n(\omega,\gamma_0)\cos(n\omega t) \right\}. \quad [1]$$

Within the linear viscoelastic regime (LVR), the response includes only the first harmonic ($n = 1$), and G'_1, G''_1 are the storage and loss moduli, respectively. Nonlinear responses are signified by the presence of higher harmonics, which increase with strain and manifest in the stress response, producing unique FT spectra with peak intensities at odd harmonics (Fig. S4). These can be interpreted using higher harmonics coefficients G'_n and G''_n, providing a detailed view of the material nonlinearities beyond the first harmonic information adopted in the correlation mode (1).

Although FT rheology is sensitive in detecting the nonlinear response, it lacks a physical interpretation of these higher harmonics. Ewoldt et al. (2, 3) developed a framework combining FT and Chebyshev polynomials to analyze nonlinear viscoelasticity. Utilizing Chebyshev decomposition, this approach allows for a physical interpretation of the higher-order coefficients. The elastic (e_n) and viscous (v_n) Chebyshev coefficients are calculated from the Fourier coefficients as

$$e_n = G'_n(-1)^{\frac{n-1}{2}} \quad n: odd, \quad [2]$$

and

$$v_n = \frac{G''_n}{\omega} = \eta'_n \quad n: odd. \quad [3]$$

Positive and negative values for these coefficients imply different physical behaviors. A positive e_3 indicates intracycle strain stiffening, while a negative e_3 corresponds to strain softening. Similarly, positive v_3 denotes shear-thickening and negative v_3 shear-thinning. The nonlinear elastic moduli, G'_L and G'_M (Eq. 4 and 5), are derived from the Fourier and Chebyshev coefficients and based on their definition from the Lissajous–Bowditch (L–B) curve (Fig. S5A). They offer a measure of the strain stiffening ($G'_L > G'_M$) or softening ($G'_L < G'_M$) within cycles;

$$G'_L \equiv \frac{\tau}{\gamma}\Big|_{\gamma=\pm\gamma_0} = \sum_{n:odd} G'_n(-1)^{\frac{n-1}{2}} = e_1 + e_3 + \cdots, \quad [4]$$

and

$$G'_M \equiv \left.\frac{d\tau}{d\gamma}\right|_{\gamma=0} = \sum_{n:odd} nG'_n = e_1 - 3e_3 + \cdots. \quad [5]$$

The strain stiffening ratio S, defined as

$$S \equiv \frac{G'_L - G'_M}{G'_L}, \quad [6]$$

is a nonlinearity index, approaching zero for linear responses, being positive for stiffening, and negative for softening materials.

The perfect plastic dissipation ratio, ϕ, quantifies the proximity of LAOS responses to ideal plastic yield-stress behavior, comparing the energy dissipated in a cycle (the shaded area in the L-B curve shown in Fig. S5A) to the energy that would be dissipated in an equivalent perfect plastic response (the area enclosed by the gray rectangle in Fig. S5B). ϕ approaches 1 for a perfect plastic behaviour, 0 for an elastic one, and $\frac{\pi}{4}$ for a Newtonian behavior. It can be calculated using the first-order viscous Fourier coefficient as:

$$\phi = \frac{\pi\gamma_0 G''_1}{4\tau_{max}}. \quad [7]$$

Our FT analysis is validated by comparing the first harmonic moduli, G'_1 and G''_1, and the stress, τ, calculated from the Fourier coefficients against those obtained by the TRIOS software in correlation mode, as illustrated in Fig. S6 for PF127. The agreement is excellent, and for conciseness, we do not include this comparison for the other samples containing HPAA additives. Across all HPAA concentrations, no notable differences are observed in the LVR width, where nonlinearity measures $\frac{e_3}{e_1}$, $\frac{v_3}{1}$, and S are approximately zero. As the strain amplitude (γ_0) increases beyond the LVR, the elastic nonlinearity measure $\frac{e_3}{e_1}$ (Fig. S7A) and the strain stiffening ratio S (Fig. 2E, main article) become positive and rise sharply, signifying strain-stiffening behavior. Conversely, $\frac{v_3}{v_1}$ (Fig. S7B) initially increases, indicating shear-thickening, before decreasing at higher strain amplitudes, suggesting a transition from shear-thickening to shear-thinning as the amplitude increases. Observations of the perfect plastic dissipation ratio, ϕ, suggest nearly purely elastic behavior at small amplitudes, where ϕ is close to zero, transitioning to ideal plastic behavior at large amplitudes as ϕ approaches unity (Fig. 2F, main article).

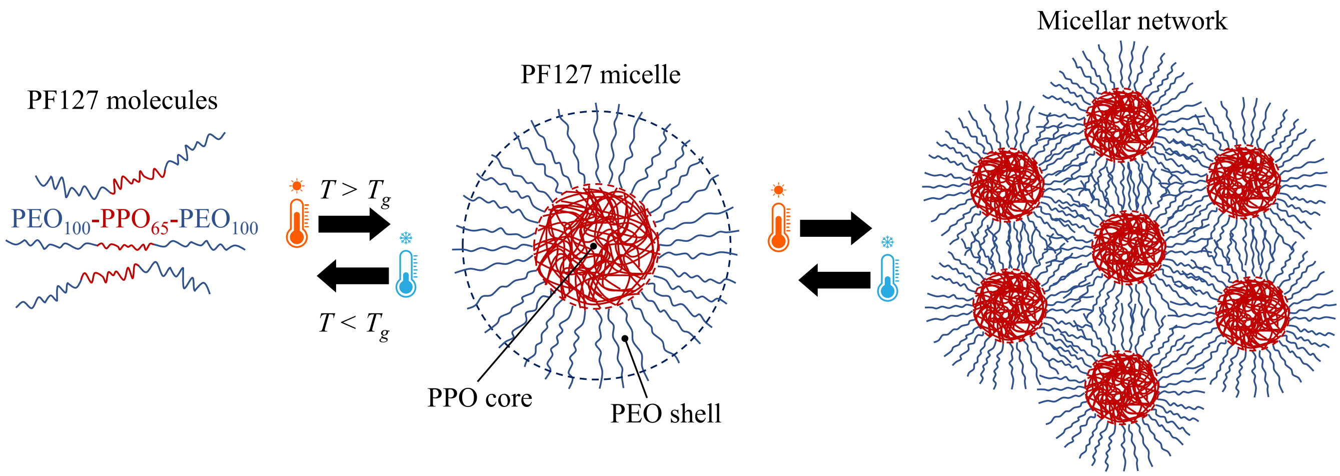

Fig. S1. Schematic representation of the thermal responsive gelation mechanism of PF127.

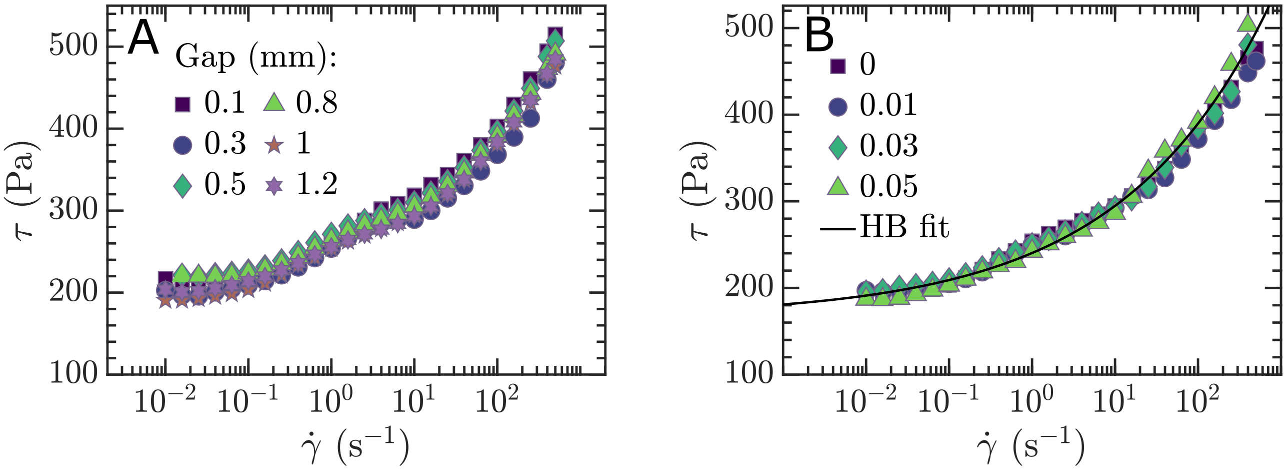

Fig. S2. Steady-state flow curves obtained using parallel plate geometry: (A) Pure PF127 with 0 wt.% HPAA additives, tested at various gap sizes, and (B) PF127 with varying concentrations of HPAA additives (indicated in the legend), each measured with a 1 mm gap. The solid line represents a fit to the Herschel-Bulkley model that gives $\tau_y = 167.1$ Pa, $k = 73$ Pa s^n and $n = 0.24$

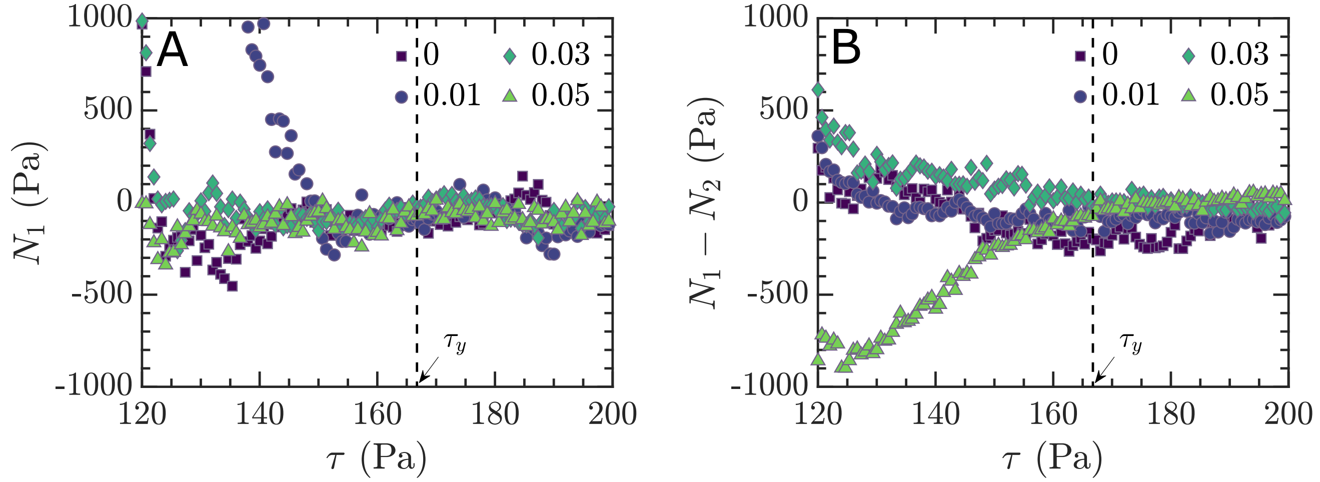

Fig. S3. Normal stress differences. (A) First normal stress difference (N_1) measured using a 40 mm and 1° cone and plate geometry. (B) Difference between the first and second normal stress differences ($N_1 - N_2$) measured using a 40 mm parallel plate geometry with a 1 mm gap. Both are plotted as functions of shear stress near the yield point. Samples were presheared at 0.01 s^{-1} for 5 minutes, allowed to rest for 20 minutes, then subjected to a stress ramp from 120 to 200 Pa at a rate of 1 Pa/min. The vertical dashed line indicates the yield stress ($\tau_y = 167.1$ Pa), derived from parallel plate measurements with a 1 mm gap as shown in Fig. S2B. At the yield point, the $N_{1,y}$ and $N_{2,y}$ values obtained from these measurements are -100, -63, -46, 6 and 119, 94, -2, 3 for 0, 0.01, 0.03, and 0.05 wt.% HPAA additives respectively. The numbers in the legend denote the weight percentages of HPAA additives.

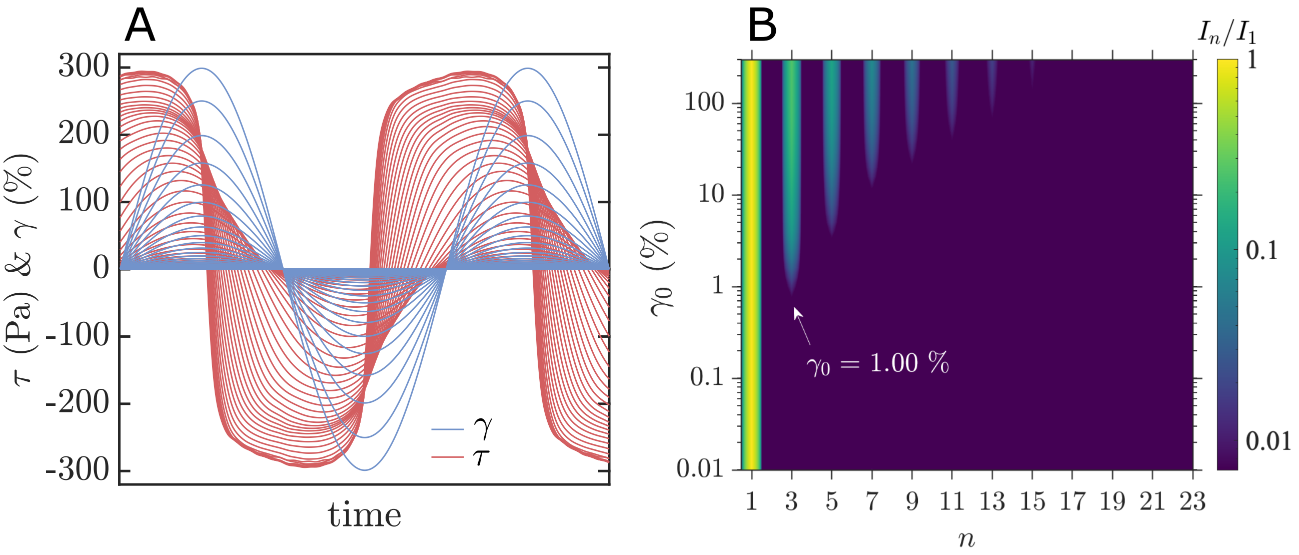

Fig. S4. Fourier transform analysis of stress response in pure PF127. (A) Stress response (red) to sinusoidal strain input (blue) across the amplitude sweep, showing distortion at high strain amplitudes due to higher harmonic contributions. (B) Contour plot illustrating the relative intensity of odd harmonics I_n/I_1 from the Fourier analysis. The arrow marks the strain amplitude γ_0 at which nonlinearities become significant, indicated by I_3/I_1 exceeding 0.01.

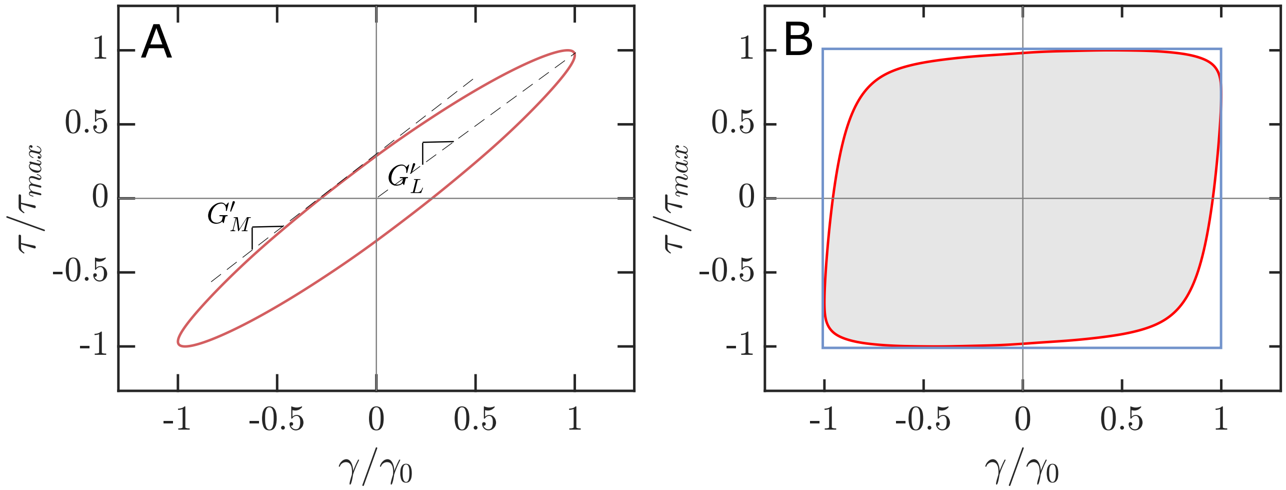

Fig. S5. Geometric interpretation of nonlinear elastic moduli via Lissajous–Bowditch curves. (A) Geometric illustration defining the nonlinear elastic moduli, G'_L and G'_M, (Eq. 4 and 5). (B) Shaded area illustrates the energy dissipated within a LAOS cycle, while the blue rectangle represents the energy dissipation expected from a perfect plastic material under identical strain conditions.

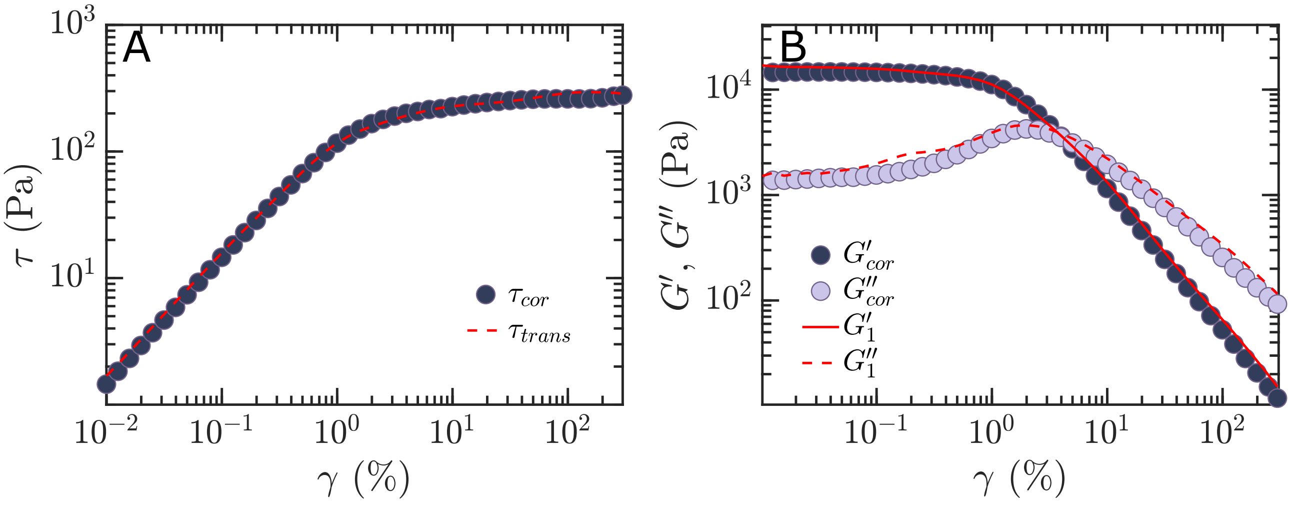

Fig. S6. Validation of Fourier transform rheology analysis for pure PF127. (A) Stress amplitude versus strain amplitude from correlation (cor, symbol) and transient (trans, dashed line) data. (B) Comparison of the first harmonic moduli, G'_1 (solid line) and G''_1 (dashed line), calculated from Fourier coefficients with the corresponding values obtained from correlation mode (cor, symbol).

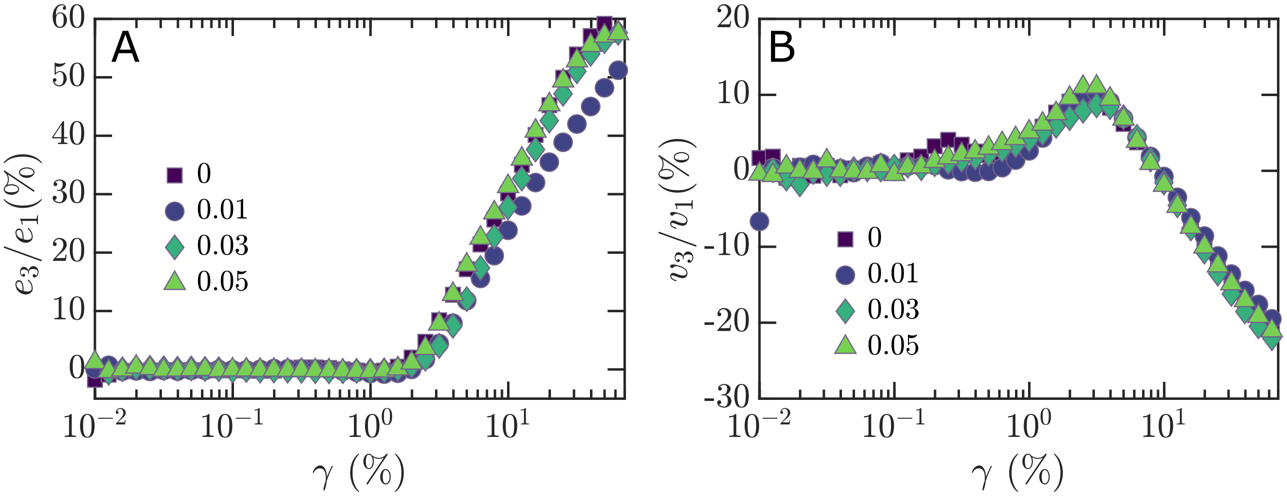

Fig. S7. Nonlinear rheological behavior from Fourier and Chebyshev analysis. (A) Ratio of elastic third harmonic Chebyshev coefficient to the first harmonic e_3/e_1, showcasing the onset and magnitude of strain stiffening behavior. (B) Ratio of viscous third harmonic Chebyshev coefficient to the first harmonic v_3/v_1, highlighting shear thickening transitioning to shear thinning with increasing strain amplitude.

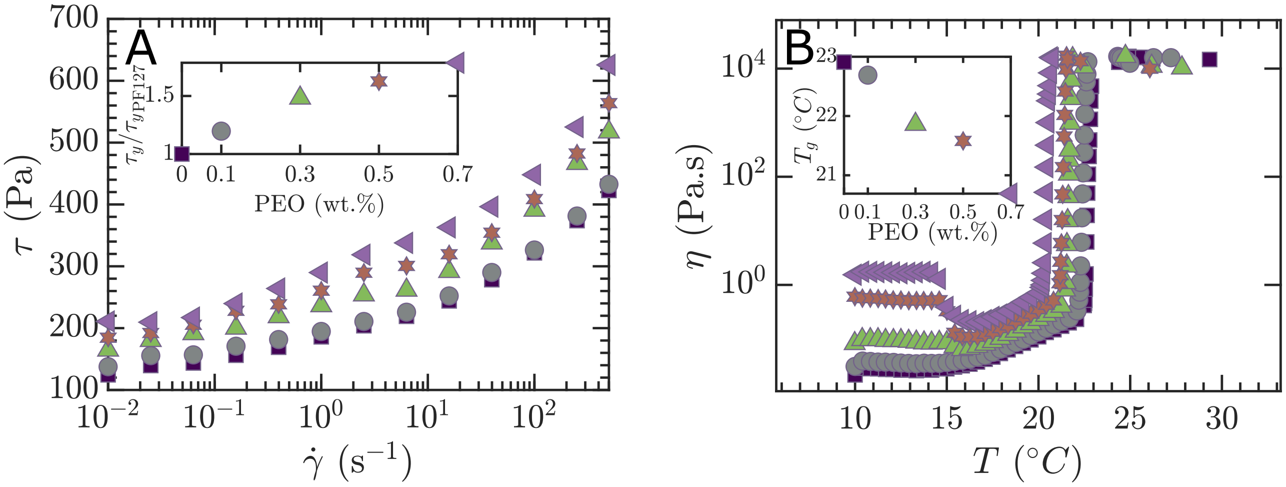

Fig. S8. Shear rheology of PF127 with PEO additives. (A) Steady-state flow curves at different PEO concentrations, with the inset highlighting the yield stress τ_y variation as a function of PEO concentration. (B) Temperature ramp tests conducted at a constant shear stress of 1 Pa and a heating rate of 1°C/min, showing the effect of PEO concentration on the gelation temperature (T_g); the inset presents the resulting shifts in T_g.

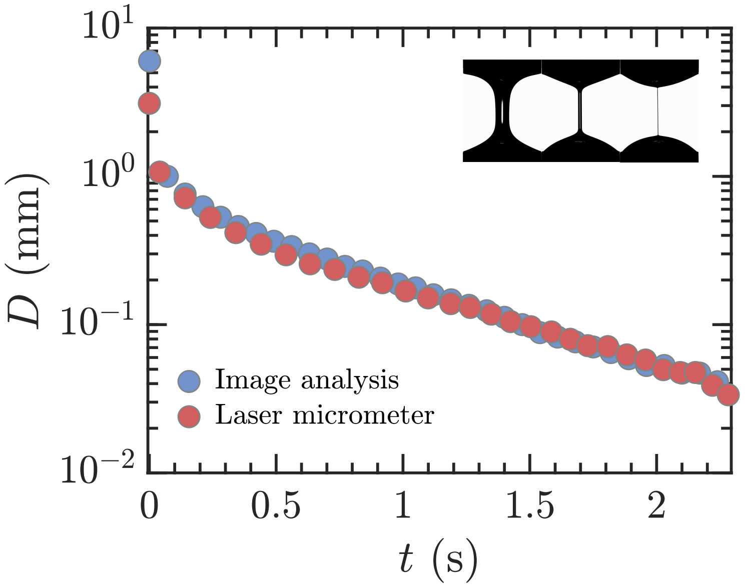

Fig. S9. Validation of our optical measurement system for CaBER analysis. The time evolution of the filament diameter of a 0.5 wt.$\%$ PEO viscoelastic solution as measured by the built-in laser micrometer of the CaBER against the filament diameter obtained through high-resolution image analysis. The inset presents a sequential time-lapse of the filament thinning captured by our optical system and used in the image analysis.

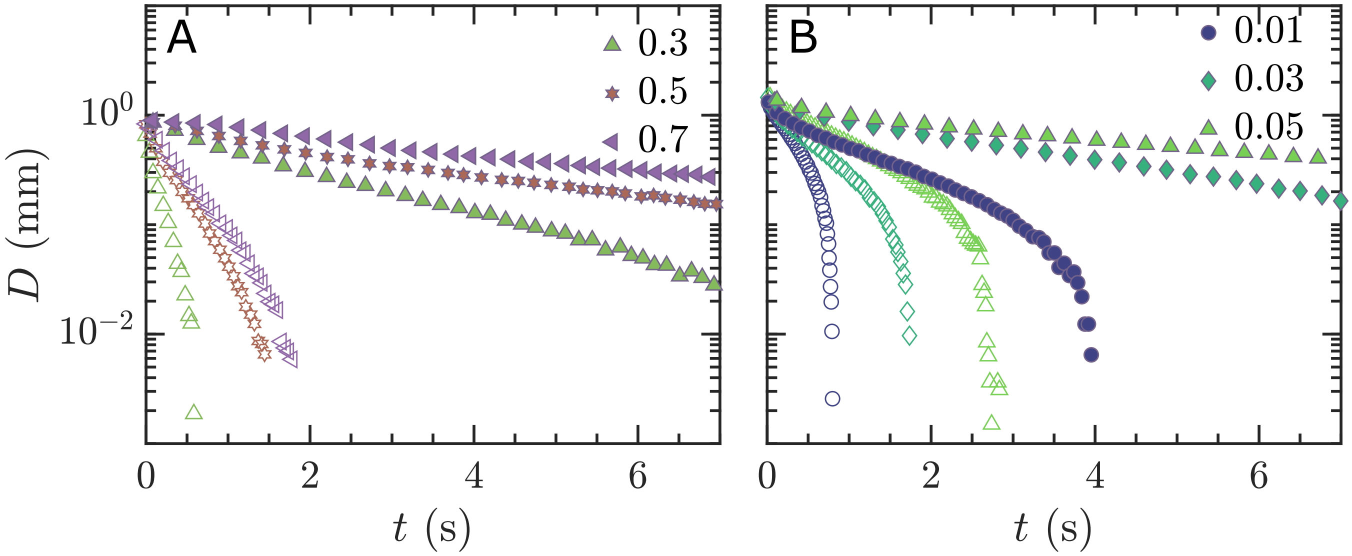

Fig. S10. Time evolution of the filament diameter obtained by the laser micrometer built-in CaBER device at $10^{\circ}C$ for (A) PEO solutions (empty symbols) and PF127 with PEO additives (filled symbols), and (B) HPAA solutions (empty symbols) and PF127 with HPAA additives (filled symbols). The relaxation times derived from these measurements are summarized in Table S1.

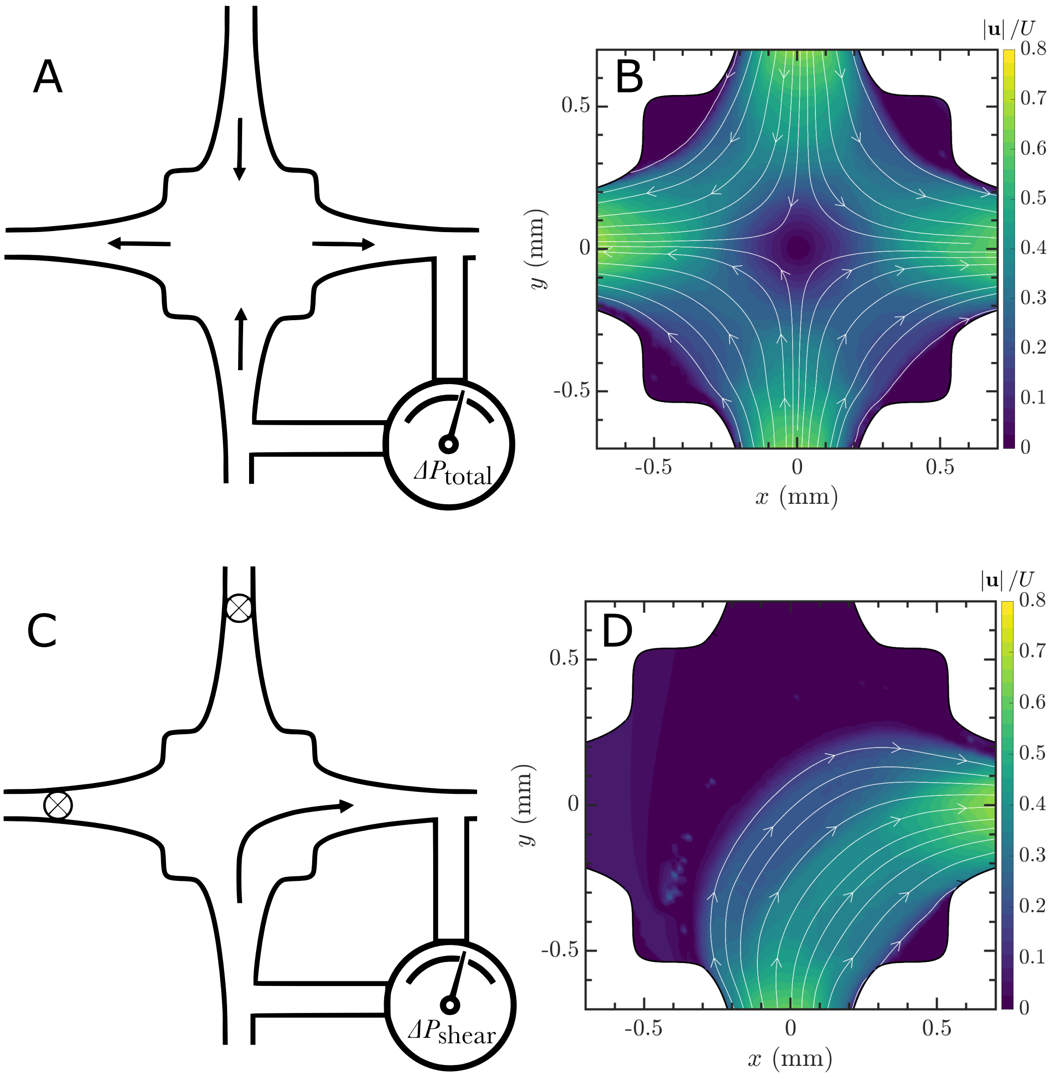

Fig. S11. Pressure difference measurements in the OSCER device. (A) Schematic representation of the total pressure difference ΔP_{total} measurement setup with all channels operational under planar elongational flow, maintaining the central stagnation point. (B) Velocity field captured during ΔP_{total} measurement. (C) Schematic of shear stress-related pressure difference ΔP_{shear} setup, with only one pair of inlet and outlet channels in use, eliminating the stagnation point. (D) Velocity field observed during ΔP_{shear} measurement. Velocity fields in (B) and (D) correspond to pure PF127 at a flow rate of 1.6 mL/min.

Table S1. Summary of relaxation times. The asterisk ($*$) refers to the measurements obtained through image analysis. Note that HPAA additives show similar relaxation times before and after gelation, due to their stable network that minimally interacts with PF127. In contrast, PEO additives exhibit variable relaxation times due to their dynamic interaction with PF127, affecting rheological properties and network stability post-gelation.

Additive (add.)	(wt.%)	add. solution (10°C)	PF127+ add. (10°C)	PF127+ add. (26°C)*
PEO	0.3	56 ms	797 ms	10.53 $\pm$ 0.4 ms
	0.5	131 ms	1.62 s	48.6 $\pm$ 1.4 ms
	0.7	159 ms	2.35 s	65.1 $\pm$ 2.9 ms
HPAA	0.01	110 ms	443 ms	316 $\pm$ 21 ms
	0.03	232 ms	1.26 s	1.99 $\pm$ 0.62 s
	0.05	330 ms	2.19 s	2.68 $\pm$ 0.36 s

Movie S1. Time-dependent behavior of EVP fluid, showing the emergence of asymmetric flow fields and the change of the flow's preferential direction over time. The transient velocity fields correspond to PF127 with a 0.05 wt.% HPAA additive at an extensional rate of 52.1 s^{-1}.

SI References

1. E García-Tuñón, R Agrawal, B Ling, DJC Dennis, Fourier-transform rheology and printability maps of complex fluids for three-dimensional printing. *Phys. Fluids* **35** (2023).
2. RH Ewoldt, AE Hosoi, GH McKinley, New measures for characterizing nonlinear viscoelasticity in large amplitude oscillatory shear. *J. Rheol.* **52**, 1427–1458 (2008).
3. RH Ewoldt, P Winter, J Maxey, GH McKinley, Large amplitude oscillatory shear of pseudoplastic and elastoviscoplastic materials. *Rheol. Acta* **49**, 191–212 (2010).
